# Intimate partner violence and mental health in Bolivia

**DOI:** 10.1186/1472-6874-13-28

**Published:** 2013-06-26

**Authors:** Dominique Meekers, Sarah C Pallin, Paul Hutchinson

**Affiliations:** 1 Tulane University, School of Public Health and Tropical Medicine, 1440 Canal Street, Suite 2200, New Orleans, LA 70112, USA; 2 Department of Sociology, Universiteit Gent, Korte Meer 3, 9000, Gent, Belgium

**Keywords:** Domestic violence, Intimate partner violence, Psychological abuse, Mental health, Bolivia, Latin America

## Abstract

**Background:**

Latin America has among the highest rates of intimate partner violence. While there is increasing evidence that intimate partner violence is associated with mental health problems, there is little such research for developing countries. The purpose of this paper is to examine the relationship between Bolivian women’s experiences with physical, psychological, and sexual intimate partner violence and mental health outcomes.

**Methods:**

This study analyzes data from the 2008 Bolivia Demographic and Health Survey. 10,119 married or cohabiting women ages 15–49 are included in the analysis. Probit regression models are used to assess the association between intimate partner violence and mental health, after controlling for other demographic factors and partner characteristics. The questionnaire uses selected questions from the SRQ-20 to measure symptoms of mental health problems.

**Results:**

Intimate partner violence is common in Bolivia, with 47% of women experiencing some type of spousal abuse in the 12 months before the survey. Women exposed to physical spousal violence in the past year are more likely to experience symptoms of depression, anxiety, psychogenic non-epileptic seizures, and psychotic disorders, after controlling for other demographic and partner characteristics. Women who experienced sexual abuse by a partner are most likely to suffer from all mental health issues. Psychological abuse is also associated with an increased risk of experiencing symptoms of depression, anxiety, and psychogenic seizures. Women who experienced only psychological abuse report mental health problems similar to those who were physically abused.

**Conclusion:**

This study demonstrates an urgent need for research on the prevalence and health consequences of psychological abuse in developing countries. Our findings highlight the need for mental health services for victims of intimate partner violence. Because physical and psychological violence are often experienced concurrently, it is recommended that health providers who are treating victims of physical intimate partner violence also screen them for symptoms of potential mental health problems and refer them to appropriate mental health services.

## Introduction

There is increasing awareness that intimate partner violence (IPV) affects all societies. However, the prevalence of IPV, including physical, sexual, and psychological violence, varies considerably. Latin America, including Bolivia, has above-average rates of domestic violence [1-5]. A substantial body of research shows that domestic violence is associated with physical health problems [1,2,5-9]. While there is ample research on the potential effects of interpersonal violence on mental health in developed countries [10-15] studies on developing countries are scarce [16,17]. This paper analyzes survey data from Bolivia to examine the relationship between women’s experiences with intimate partner violence and mental health.

## Background

### Definition and prevalence of intimate partner violence

Existing studies on IPV frequently use the terms intimate partner violence, domestic abuse, domestic violence, and spousal violence interchangeably [7,18]. Spousal violence may refer to partner violence between married couples, cohabiting couples, and individuals in other forms of non-marital unions. Several definitions of domestic violence make specific reference to psychological abuse and threats of harm. The 1993 UN General Assembly defines gender-based violence as any act “that results in, or is likely to result in, physical, sexual, or psychological harm or suffering to women, including threats of such acts, coercion, or arbitrary deprivations of liberty, whether occurring in public or private life” [19]. WHO defines domestic violence as “any behavior within an intimate relationship that causes physical, psychological or sexual harm” [20].

While intimate partner violence exists in all societies, its prevalence varies widely by region [4,7,20,21]. WHO surveys show that the percentage of ever-partnered women who reported ever experiencing either physical or sexual violence ranged from 15% in some Japanese sites to over 60% in sites in Bangladesh, Ethiopia, and Peru [4,20]. Several studies show high rates of IPV in Latin America. For example, 49% of Peruvian women experienced severe physical violence in their lifetime, and 38% of women in Colombia report being physically or sexually abused by a recent partner [4,5]. A national survey of Paraguay shows that 18.4% of women who were ever in union experienced psychological IPV, 6.7% physical IPV, and 3.2% sexual IPV [16]. In Bolivia, one study found that 52% and 14% of women experienced physical and sexual violence, respectively [7], and another found that 40% had been physically or sexually abused by their spouse in the past year [22]. A recent study of the Tsimane forager-horticulturalists reports that 85% of women had experienced physical spousal abuse, and 38% experienced physical abuse in the last year [23].

The WHO surveys also asked women whether their partner had engaged in emotional abuse (e.g., insulting them, belittling or humiliating them, intimidating or scaring them, or threatening to harm them). The percentage of ever-partnered women who reported ever experiencing any such acts ranged from less than 20% in Samoan sites to 75% in Ethiopian sites. The percentage who experienced such events during the year before the survey ranged from 12% in Samoa and urban Serbia and Montenegro to 58% in Ethiopia [4,20]. The same study also revealed large variations in the percentage of ever-partnered women who reported that their partner had put constraints on their physical or social mobility (21% in urban Japan compared to nearly 90% in urban Tanzania).

Low education and socioeconomic status, being younger, and non-marital cohabitation tend to increase women’s risk of experiencing domestic violence [7,16,24-27]. Exposure to violence in the parental household – e.g. physical abuse in childhood and witnessing inter-parental abuse – also increases the risk of experiencing spousal abuse in adulthood [28-31][16]. By contrast, differences in age and education between partners typically have weak or inconsistent associations with women’s risk of domestic violence [7,24,27,32].

This study examines the association between various aspects of intimate partner violence and indicators of mental health using recent survey data from Bolivia.

#### Data

Our analysis is based on the 2008 Bolivia Demographic and Health Survey [33] which contains data on a nationally representative sample of women aged 15–49 years. The BDHS is part of the larger MEASURE Evaluation Demographic and Health Surveys Phase II project, which is funded by the United States Agency for International Development (USAID). All DHS data are publicly available ( http://www.measuredhs.com/data). The BDHS data were collected by the Bolivian Ministry of Health and Sports (Ministerio de Salud y Deportes) and National Institute for Statistics (Instituto Nacional de Estadística) with technical assistance from ORC Macro (ORC Macro has since become ICF International). The Institutional Review Board of ORC Macro reviewed and approved the MEASURE Demographic and Health Surveys Project Phase II, including the BDHS. The Institutional Review Board of ORC Macro complied with the United States Department of Health and Human Services regulations for the protection of human research subjects (45 CFR 46). Further details about the data collection are available in the survey report [33].

Informed consent was obtained from all respondents. All survey staff received special training for the implementation of the domestic violence module. During the interviews, informed consent was re-iterated before the start of the domestic violence module. Our analysis is restricted to women who are currently married or in union (n = 10,188). After eliminating cases with missing values, 10,119 respondents were retained for analysis. The BDHS questionnaire collected information on a wide range of topics, including domestic violence and mental health. (The survey questionnaire is available at http://www.measuredhs.com/publications/publication-FR228-DHS-Final-Reports.cfm).

#### Measures

The BDHS questionnaire includes nine mental health questions, seven of which are a subset of the Self Reporting Questionnaire 20 (SRQ-20), a mental health screening tool developed by WHO [34]. The SRQ-20 has been used widely to screen for the presence of non-psychotic mental health disorders [35-41]. The SRQ-20 has also been validated as a screening tool for psychological morbidity [42-45]. The BDHS also includes selected mental health questions from the extended version of the Self-Reported Questionnaire, the SRQ-25. The SRQ-25 includes four additional questions aimed at identifying probable psychosis and one question about psychogenic non-epileptic seizures, or convulsions. The SRQ-25 has also been widely used to assess the presence of mental health disorders [46-49].

The nine mental health questions in the BDHS aim to help identify four aspects of mental health: depression, anxiety, psychogenic seizures, and psychotic disorders. Since it has been reported that depression and anxiety account for most of the IPV disease burden [50], our study distinguishes between the four different aspects of mental health. To measure whether the respondent has symptoms of depression, all respondents were asked whether they feel tired all the time, whether they cry easily, whether they have difficulty performing daily activities, and whether they find it difficult to make decisions. To identify whether the respondent has symptoms of anxiety disorders, all respondents were asked whether they often have headaches of great intensity at the nape of the neck, whether they feel fear for no apparent reason, and whether they are easily frightened. In addition, one question was used to assess whether the respondent had symptoms of psychotic disorders (“Do you hear voices that talk to you and that others do not hear?”) and one question aimed to identify whether the respondent had symptoms of psychogenic non-epileptic seizures (“Have you experienced convulsions or attacks with tongue-biting and loss of consciousness?).

The domestic violence module in the BDHS questionnaire is based on the Conflict Tactics Scale (CTS) developed by Murray Straus [30,51]. Specifically, women were asked whether their current partner engaged in any acts of physical, sexual, or psychological abuse towards them during the 12 months before the survey. To measure exposure to physical abuse, women were asked how often (one time, a few times, or often) in the last 12 months their partner had pushed or pinched them, beaten or kicked them, beaten them with an object, or tried to strangle or burn them. Exposure to sexual abuse was measured by asking women whether their partner forced them to have sexual relations against their will during the year before the survey. Exposure to psychological abuse was measured by asking the respondents how often their partner, in the past 12 months, had accused them of being unfaithful, had been jealous after she talked with a man, had attempted to limit her contact with her family, had humiliated or insulted her, had threatened to abandon her, had threatened to take away her children, had threatened to take away economic support, or had broken things inside the house.

Our summary indicator of experience with intimate partner violence is a 4-category variable that identifies which of the three types of abuse the respondent experienced in the past year (1. No intimate partner abuse; 2. psychological violence *only*; 3. physical abuse or both physical and psychological abuse; 4. any sexual abuse).

Our control variables include the respondent’s socioeconomic and demographic status, her partner’s socioeconomic and demographic status, and exposure to domestic abuse in the respondent’s parental household. Respondent characteristics include her age group, place of residence (rural vs. urban), marital status (married vs. cohabiting), region of residence, employment status, and socioeconomic status. The respondent’s socioeconomic status was assessed using the DHS wealth index, which uses information on household assets, services and amenities to classify respondents into wealth quintiles [52].

Controls for the partner’s characteristics include his age group, the age difference between the respondent and her partner, partner’s level of education, and the difference in the level of education between the respondent and her partner. To measure the respondent’s exposure to domestic abuse in her parental household, we include dichotomous variables that measure whether she was physically abused in childhood (yes/no), whether she was psychologically abused as a child (yes/no), and whether her father beat her mother (no vs. yes/don’t know). Women who reported being punished as a child by being beaten, slapped, having their ears pulled, having food withheld, or having water thrown on them were coded as having experienced physical abuse. Women who reported punishment by being shouted at, insulted, locked up, left outside, having their clothes taken away, or being ignored for more than a day were coded as having experienced psychological abuse in childhood.

## Methods

We use probit regression models to examine the association between exposure to intimate partner violence and indicators of mental health, after controlling for other factors [53]. To facilitate interpretation, the probit results are converted to the predicted probability of experiencing various mental health outcomes using STATA12’s *margins* command [54]. For simplicity, we present the predicted probabilities in the form of the predicted percentage of women who experienced each mental health outcome. For each health outcome, we present two models. The first model shows the unadjusted percentage of respondents who experienced the outcome; the second model shows the adjusted percentages after controlling for other factors. In the text, we will focus on the IPV variables and childhood experience with violence. Because we cannot rule out that some women have left a relationship because of abuse, our analysis may underestimate the association between intimate partner violence on mental health.

## Results

Table 1 describes the characteristics of the working sample of married and cohabiting women. Most women lived in urban areas (61.1%), but had a relatively low level of education; less than half of women attended secondary school. As anticipated, the sample has a fairly young age distribution. Nearly two thirds of women are married (62.8%), while the remainder are cohabiting.

**Table 1:** **Sample description** (**married and cohabiting women only**)

	**% (weighted)**	**%(unweighted)**	**n**
Residence			
Urban	61.1%	58.5%	5,924
Rural	38.9	41.5	4,195
Education			
None	6.4	6.2	630
Primary	51.4	50.5	5,109
Secondary	28.3	28.0	2,829
Higher	14.0	15.3	1,551
SES			
First quintile	18.5	20.3	2,054
Second quintile	19.1	19.5	1,976
Third quintile	21.0	19.9	2,018
Fourth quintile	22.0	20.8	2,108
Fifth quintile	19.3	19.4	1,963
Respondent’s work status			
Working	66.3	64.5	6,511
Not working	33.7	35.5	3,577
Region			
La Paz	29.2	18.7	1,895
Chuquisaca	5.7	8.7	881
Cochabamba	17.7	13.0	1,315
Oruro	5.2	8.4	854
Potosí	10.2	10.6	1,079
Tarija	4.7	9.8	987
Santa Cruz	22.9	18.8	1,897
Beni	3.6	7.1	718
Pando	0.7	4.8	493
Respondent’s age			
15-24	17.2	18.1	1,831
25-34	38.5	37.7	3,816
35-44	32.0	31.9	3,231
45-49	12.4	12.3	1,241
Respondent’s marital status			
Married	62.8	61.1	6,178
Cohabiting	37.2	39.0	3,941
Partner’s age			
14-24	10.0	10.2	1,029
25-34	34.2	34.0	3,439
35-44	33.6	33.2	3,353
45+	22.2	22.7	2,290
Partner’s age, relative to respondent			
Same age or younger	29.0	28.2	2,851
1-5 years older	44.3	44.2	4,465
6 or more years older	26.7	27.6	2,795
Partner’s education			
None	1.5	1.8	181
Primary	44.0	44.5	4,502
Secondary	35.9	33.9	3,429
Higher	18.5	19.8	1,997
Education differences between partners			
Wife is more educated	21.6	23.2	5,348
Same level of education	23.4	24.0	2,425
Husband is more educated	55.0	52.8	2,334
Respondent experienced physical abuse in childhood			
No	28.3	29.0	2,930
Yes	71.7	71.0	7,184
Respondent experienced psychological abuse in childhood			
No	57.6	59.7	6,033
Yes	42.4	40.4	4,081
Respondent reports father beat mother			
No	46.1	48.1	5,250
Yes/Don’t know	53.9	51.9	4,860
Respondent’s experience with intimate partner violence in last 12 months			
None	52.8	53.0	5,365
Psychological abuse only	21.1	20.8	2,106
Physical abuse (no sexual)	19.2	19.1	1,933
Sexual abuseΨ	6.9	7.1	715
Total	100%	100%	10,119

Ψ Most victims of sexual abuse also experienced psychological and/or physical abuse.

Nearly half of all women (44.3%) reported that their partner was one to five years older than themselves, while 26.7% reported that their partner was at least six years older. On average, men have somewhat higher levels of education than women. In over half of the couples (55.0%), the male partner has the highest level of education. Nevertheless, one in five women (21.6%) report having a higher level of education than their partner.

The third panel of Table 1 shows information about domestic abuse in the respondent’s parental household. Overall, 71.7% of women experienced physical abuse as a child, and 42.4% experienced psychological abuse. When respondents were asked whether their father beat their mother, only 46.1% denied that this happened. The rest either admitted that their father beat their mother or reported not knowing whether this happened.

Overall, 47.2% of women reported experiencing some type of intimate partner violence in the past year: 21.1% experienced only psychological abuse, and 19.2% experienced both physical abuse or both physical and psychological abuse. In addition, 6.9% were sexually abused by their partner. Most victims of sexual abuse by their partner were also physically or psychologically abused (82.8% and 94.8%, respectively, not shown).

Tables 2 and 3 show the predicted percentage of women who reported having experienced various physical and mental health outcomes, by type of intimate partner violence experienced. Table 2 shows indicators of depression; Table 3 shows indicators anxiety, psychotic disorders, and psychogenic non-epileptic seizures. For both tables, two models are shown for each indicator. The first model shows the unadjusted percentage of women who report experiencing the symptom; the second model shows the adjusted percentage after controlling for other factors.

**Table 2:** **Unadjusted and adjusted percentage of women experiencing various symptoms of depression**, **by exposure to intimate partner violence**, **socioeconomic and demographic characteristics of the respondent**, **partner**’**s characteristics**, **and exposure to domestic abuse in the parental household**

	**Symptoms of depression**							
	**Feels tired all the time**		**Cries easily**		**Difficulty doing daily activities**		**Difficulty making decisions**	
	**(1)**	**(2)**	**(1)**	**(2)**	**(1)**	**(2)**	(**1**)	**(2)**
	**Unadjusted**	**Adjusted**	**Unadjusted**	**Adjusted**	**Unadjusted**	**Adjusted**	**Unadjusted**	**Adjusted**
	**Percentage**	**Percentage**	**Percentage**	**Percentage**	**Percentage**	**Percentage**	**Percentage**	**Percentage**
	**(CI)**	**(CI)**	**(CI)**	**(CI)**	**(CI)**	**(CI)**	**(CI)**	**(CI)**
Intimate partner violence								
No abuse (reference)	49.8	49.9	72.1	72.7	28.0	28.0	42.1	42.7
	(48.1-51.6)	(48.2-51.6)	(70.6-73.7)	(71.2-74.2)	(26.3-29.7)	(26.4-29.7)	(40.3-43.8)	(41.0-44.4)
Psychol. abuse only	55.9***	57.5***	74.6	74.2	33.6***	34.0***	50.8***	50.8***
	(53.6-58.3)	(55.2-59.8)	(72.2-77.0)	(71.8-76.6)	(31.3-35.9)	(31.7-36.3)	(48.2-53.3)	(48.2-53.4)
Physical abuse (no sexual)	56.3***	55.5**	79.4***	78.6***	34.1***	34.1***	51.9***	50.7***
	(53.6-58.9)	(52.9-58.1)	(77.1-81.7)	(76.3-80.9)	(31.4-36.7)	(31.4-36.8)	(49.1-54.7)	(47.9-53.6)
Sexual abuse¥	68.3***	66.4***	86.3***	85.9***	43.8***	42.4***	63.8***	62.4***
	(64.0-72.5)	(62.2-70.6)	(83.1-89.6)	(82.4-89.3)	(39.0-48.7)	(37.6-47.1)	(59.5-68.2)	(58.2-66.6)
Respondent experienced physical abuse in childhood								
No (reference)		52.3		72.1		31.0		45.9
		(50.1-54.5)		(70.1-74.0)		(28.7-33.3)		(43.5-48.3)
Yes		54.3		76.2***		31.6		47.9
		(52.9-55.7)		(75.0-77.5)		(30.2-33.1)		(46.4-49.4)
Respondent experienced psychological abuse in childhood								
No (reference)		55.4		75.7		31.9		48.7
		(54.0-56.9)		(74.3-77.1)		(30.2-33.6)		(47.0-50.3)
Yes		51.4**		74.1		30.8		45.6*
		(49.5-53.4)		(72.4-75.8)		(29.0-32.7)		(43.5-47.6)
Respondent reports father beat mother								
No (reference)		53.3		73.2		30.9		44.1
		(51.5-55.1)		(71.6-74.8)		(29.3-32.5)		(42.3-45.9)
Yes/Don’t know		54.1		76.7**		31.9		50.1***
		(52.4-55.8)		(75.3-78.0)		(30.2-33.6)		(48.4-51.8)
Region								
La Paz (reference)		62.3		78.9		34.6		51.8
		(59.8-64.7)		(76.6-81.2)		(31.9-37.4)		(49.3-54.3)
Chuquisaca		44.8***		80.2		24.8***		48.3
		(39.6-50.0)		(77.3-83.0)		(20.6-29.0)		(44.3-52.3)
Cochabamba		57.8*		77.0		35.6		47.0*
		(54.8-60.9)		(74.7-79.4)		(32.3-39.0)		(43.7-50.4)
Oruro		49.0***		73.5**		27.2**		47.3*
		(45.3-52.6)		(70.2-76.8)		(23.5-31.0)		(43.5-51.0)
Potosí		54.5***		65.3***		27.4**		43.8**
		(51.0-57.9)		(61.6-68.9)		(23.6-31.3)		(38.5-49.1)
Tarija		53.1***		73.7*		38.1		55.2
		(49.6-56.6)		(70.2-77.2)		(34.9-41.3)		(51.6-58.7)
Santa Cruz		45.9***		73.2**		28.8**		44.0***
		(43.1-48.7)		(70.6-75.9)		(26.3-31.4)		(41.8-46.2)
Beni		33.9***		70.5***		21.3***		32.3***
		(28.1-39.6)		(66.8-74.1)		(17.5-25.1)		(27.0-37.6)
Pando		54.2**		66.8***		31.3		46.5
		(48.8-59.5)		(61.9-71.8)		(26.4-36.2)		(39.4-53.5)
Residence								
Urban (reference)		54.6		75.1		31.5		47.4
		(52.5-56.6)		(73.4-76.9)		(29.4-33.5)		(45.3-49.4)
Rural		52.4		74.8		31.4		47.3
		(49.5-55.3)		(72.5-77.2)		(28.5-34.3)		(46.2-50.4)
Education								
None (reference)		57.7		76.2		34.0		45.3
		(52.1-63.3)		(71.3-81.2)		(28.9-39.2)		(39.6-51.0)
Primary		57.8		74.4		33.5		49.7
		(55.7-59.9)		(72.5-76.3)		(31.6-35.5)		(44.1-48.9)
Secondary		50.2*		77.2		28.9		47.7
		(47.6-52.9)		(74.9-79.4)		(26.4-31.4)		(45.0-50.4)
Higher		44.1**		72.4		26.9		38.3
		(39.4-48.9)		(68.2-76.7)		(22.8-31.0)		(33.5-43.1)
SES								
First quintile (reference)		57.4		72.6		30.4		45.1
		(53.1-61.7)		(69.2-76.1)		(26.6-34.2)		(41.0-49.2)
Second quintile		55.2		74.4		34.1		46.6
		(51.7-58.8)		(71.6-77.3)		(30.8-37.3)		(43.6-49.7)
Third quintile		55.7		75.9		35.3		50.0
		(53.0-58.3)		(73.6-78.3)		(32.4-38.1)		(47.3-52.6)
Fourth quintile		53.1		78.4*		33.0		50.6
		(50.2-56.1)		(75.8-80.9)		(30.0-36.0)		(47.5-53.8)
Fifth quintile		47.4**		73.2		23.6*		43.4
		(43.5-51.2)		(69.9-76.6)		(20.2-26.9)		(39.9-47.0)
Work status								
Not working (reference)		51.6		75.1		29.8		46.5
		(49.5-53.7)		(73.3-76.9)		(27.8-31.8)		(44.3-48.6)
Working		54.8		75.0		32.3		47.8
		(53.3-56.3)		(73.6-76.3)		(30.7-33.8)		(46.3-49.3)
Age								
15-24 (reference)		48.1		72.0		27.9		49.2
		(43.8-52.3)		(67.7-76.2)		(23.9-32.0)		(44.6-53.8)
25-34		52.1		74.5		30.3		46.5
		(50.0-54.3)		(72.5-76.6)		(28.2-32.5)		(44.1-48.9)
35-44		56.6**		75.8		32.7		46.3
		(53.9-59.3)		(73.4-78.1)		(30.0-35.3)		(43.6-49.1)
45-49		59.5**		78.5		36.4*		50.0
		(55.0-63.9)		(74.5-82.5)		(31.6-41.1)		(44.8-55.3)
Marital status								
Married (reference)		54.8		75.2		30.9		46.8
		(53.3-56.4)		(73.8-76.7)		(29.4-32.5)		(45.2-48.4)
Cohabiting		51.9*		75.0		32.4		48.3
		(49.8-54.1)		(72.5-76.7)		(30.3-34.4)		(46.2-50.4)
Partner’s age								
14-24 (reference)		54.1		76.6		32.4		48.0
		(48.7-59.5)		(72.2-80.9)		(27.4-37.4)		(42.4-53.6)
25-34		54.0		76.2		28.4		46.2
		(51.4-56.6)		(74.0-78.5)		(25.8-31.0)		(43.5-49.0)
35-44		53.5		74.0		31.7		48.4
		(51.4-55.7)		(71.9-76.1)		(29.6-33.9)		(46.0-50.8)
45+		53.5		73.9		35.2		47.1
		(49.6-57.4)		(70.2-77.7)		(31.4-39.0)		(42.9-51.3)
Partner’s age, relative to respondent								
Same age or younger (ref.)		55.4		76.7		33.2		47.7
		(52.8-58.0)		(74.5-78.8)		(30.8-35.6)		(45.2-50.2)
1-5 yrs. older		53.1		74.2		31.2		47.2
		(51.4-54.7)		(72.5-75.8)		(29.5-33.0)		(45.4-49.0)
6+ yrs. older		53.1		74.7		29.9		47.1
		(50.4-55.7)		(72.4-77.0)		(27.4-32.4)		(44.5-49.8)
Partner’s education								
None (reference)		52.6		75.4		20.3		41.7
		(42.9-62.4)		(67.8-82.9)		(12.5-28.0)		(32.4-51.0)
Primary		55.7		75.4		32.0**		47.1
		(53.3-58.1)		(73.3-77.5)		(29.9-34.2)		(44.7-49.6)
Secondary		55.0		75.2		32.1*		49.3
		(52.9-57.2)		(73.2-77.3)		(30.2-34.0)		(47.2-51.5)
Higher		46.6		73.8		29.6		44.3
		(42.5-50.6)		(70.4-77.2)		(26.0-33.2)		(40.3-48.3)
Education difference between partners								
Wife is more educated (ref.)		53.5		73.9		31.1		46.5
		(50.7-56.3)		(71.2-76.5)		(28.4-33.8)		(43.4-49.5)
Same level of education		50.3		73.9		29.0		46.1
		(47.7-52.9)		(71.4-76.4)		(26.7-31.2)		(43.7-48.6)
Husband is more educated		55.3		73.8		32.6		48.2
		(53.4-57.2)		(74.3-77.6)		(30.7-34.5)		(46.2-50.1)

*p < 0.05 **p < 0.01 ***p < 0.001.

Ψ Respondents who experienced sexual abuse may also have experienced psychological and/or physical abuse.

**Table 3:** **Unadjusted and adjusted percentage of women who experienced various symptoms of anxiety**, **symptoms of psychogenic non**-**epileptic seizures**, **and of psychotic disorders**, **by exposure to intimate partner violence**, **socioeconomic and demographic characteristics of the respondent**, **partner**’**s characteristics**, **and exposure to domestic abuse in the parental household**

	**Symptoms of anxiety**						**Symptoms of psychogenic non**-**epileptic seizures**		**Symptoms of psychotic disorders**	
	**Feelings of fear for no apparent reason**		**Scares easily**		**Headaches of great intensity**		**Convulsions**, **attacks with tongue biting**		**Hears voices that others do not hear**	
	**(1)**	**(2)**	**(1)**	**(2)**	**(1)**	**(2)**	**(1)**	**(2)**	**(1)**	**(2)**
	**Unadjusted**	**Adjusted**	**Unadjusted**	**Adjusted**	**Unadjusted**	**Adjusted**	**Unadjusted**	**Adjusted**	**Unadjusted**	**Adjusted**
	**Percentage**	**Percentage**	**Percentage**	**Percentage**	**Percentage**	**Percentage**	**Percentage**	**Percentage**	**Percentage**	**Percentage**
	**(CI)**	**(CI)**	**(CI)**	**(CI)**	**(CI)**	**(CI)**	**(CI)**	**(CI)**	**(CI)**	**(CI)**
Intimate partner violence										
No abuse (reference)	45.6	46.0	62.8	63.3	51.0	50.8	6.5	6.4	11.2	11.5
	(43.9-47.3)	(44.3-47.7)	(61.1-64.6)	(61.6-65.0)	(49.3-52.7)	(49.1-52.4)	(5.7-7.4)	(5.6-7.2)	(10.1-12.3)	(10.4-12.7)
Psychol. abuse only	49.0*	49.7*	67.0**	67.0*	58.5***	58.9**	8.3*	8.6**	13.9*	13.5
	(46.4-51.6)	(50.8-56.2)	(64.7-69.4)	(64.6-69.4)	(55.9-61.1)	(56.4-61.5)	(6.7-9.8)	(7.0-10.2)	(12.0-15.7)	(11.7-15.3)
Physical abuse (no sexual)	54.9***	53.5***	67.6**	66.6	58.0***	58.3***	9.3**	9.4***	15.7***	15.3**
	(52.1-57.7)	(50.8-56.2)	(64.9-70.3)	(63.9-69.4)	(55.3-60.7)	(55.6-60.9)	(7.7-10.8)	(7.9-11.0)	(13.4-17.9)	(13.0-17.5)
Sexual abuse¥	61.2***	59.3***	72.8***	71.8***	66.9***	65.5***	13.1***	12.4***	25.2***	24.0***
	(56.9-65.5)	(55.1-63.7)	(68.5-77.1)	(67.6-76.0)	(62.4-71.4)	(61.1-69.9)	(9.9-16.4)	(9.2-15.6)	(21.0-29.3)	(20.1-27.9)
Respondent experienced physical abuse in childhood										
No (reference)		47.2		64.0		52.1		7.0		12.2
		(44.9-50.9)		(61.8-66.1)		(49.9-54.4)		(5.7-8.3)		(10.6-13.8)
Yes		49.9		65.9		56.1**		8.2		14.1
		(48.4-51.4)		(64.4-67.3)		(54.7-57.5)		(7.4-9.0)		(13.0-15.2)
Respondent experienced psychological abuse in childhood										
No (reference)		49.3		64.8		55.3		8.0		14.0
		(47.7-50.9)		(63.2-66.4)		(53.7-56.9)		(7.1-8.9)		(12.8-15.2)
Yes		49.0		66.0		54.5		7.7		13.0
		(47.1-50.9)		(64.2-67.8)		(52.6-56.3)		(6.7-8.7)		(11.7-14.3)
Respondent reports father beat her mother										
No (reference)		48.4		64.3		54.2		7.0		12.7
		(46.6-50.2)		(62.6-66.0)		(52.3-56.0)		(6.1-8.0)		(11.3-14.1)
Yes/Don’t know		49.8		66.2		55.6		8.5*		14.3
		(48.0-51.2)		(64.6-67.8)		(53.9-57.4)		(7.5-9.5)		(13.1-15.4)
Region										
La Paz (reference)		53.3		69.9		54.3		6.9		13.5
		(50.5-56.1)		(67.6-72.3)		(51.9-56.8)		(5.6-8.1)		(11.4-15.5)
Chuquisaca		43.0**		60.7***		54.5		5.2		6.7***
		(37.9-48.2)		(56.4-65.0)		(48.0-61.0)		(3.7-6.8)		(4.7-8.7)
Cochabamba		50.8		66.4*		61.0**		11.3***		15.2
		(48.1-53.6)		(63.3-69.5)		(58.0-64.0)		(9.0-13.7)		(13.3-17.2)
Oruro		49.0		63.3**		50.1		6.5		6.8***
		(44.5-53.4)		(59.5-67.2)		(44.6-55.6)		(4.7-8.3)		(4.8-8.8)
Potosí		46.1		58.6***		45.8***		7.7		10.7
		(41.5-50.6)		(54.6-62.5)		(42.2-49.3)		(5.5-9.7)		(8.2-13.2)
Tarija		51.9		68.5		58.3		7.6		17.5*
		(48.6-55.2)		(64.6-72.4)		(55.2-61.4)		(5.5-9.7)		(14.5-20.5)
Santa Cruz		45.6***		64.8**		55.0		6.7		16.1
		(43.0-48.1)		(61.8-67.7)		(52.1-57.9)		(5.4-8.0)		(13.8-18.4)
Beni		46.3*		51.3***		58.6		11.6**		12.2
		(41.0-51.7)		(45.9-56.6)		(54.2-63.0)		(8.3-14.8)		(9.4-14.9)
Pando		44.3**		61.3**		59.6		12.9***		21.1**
		(39.6-49.0)		(56.5-66.1)		(54.4-64.6)		(9.4-16.3)		(16.6-25.7)
Residence										
Urban (reference)		48.5		64.9		53.1		6.9		13.7
		(46.5-50.5)		(62.8-66.9)		(50.7-55.4)		(5.8-8.0)		(12.2-15.2)
Rural		50.1		66.0		58.0		9.0		13.4
		(47.2-53.1)		(63.4-68.6)		(54.7-61.2)		(7.5-10.6)		(11.4-15.4)
Education										
None (reference)		53.1		69.4		61.1		7.5		11.3
		(47.6-58.6)		(64.4-74.4)		(55.4-66.7)		(5.1-9.9)		(7.7-14.9)
Primary		51.0		67.5		58.9		8.2		14.7
		(49.0-53.0)		(65.5-66.7)		(56.7-61.1)		(7.2-9.3)		(13.2-16.3)
Secondary		48.6		64.2		51.3**		7.5		13.1
		(46.0-51.2)		(61.7-66.7)		(48.6-54.0)		(6.0-9.0)		(11.2-14.9)
Higher		41.3**		58.2**		45.3***		6.8		10.9
		(36.3-46.4)		(53.5-62.9)		(40.4-50.3)		(4.3-9.4)		(7.5-14.3)
SES										
First (reference)		50.1		66.6		57.9		9.1		11.5
		(46.3-53.9)		(62.8-70.4)		(53.9-61.9)		(7.0-11.1)		(9.1-14.0)
Second		49.2		66.9		52.4**		7.7		14.4*
		(45.9-52.4)		(63.8-70.1)		(48.2-56.7)		(6.2-9.3)		(12.1-16.8)
Third		51.4		67.2		55.3		7.9		15.1
		(48.7-54.1)		(64.6-69.8)		(52.6-58.0)		(6.3-9.6)		(12.9-17.2)
Fourth		49.1		65.3		55.2		6.5		14.3
		(46.2-52.0)		(62.5-68.0)		(52.1-58.4)		(4.9-8.2)		(12.3-16.3)
Fifth		45.8		60.8		54.0		7.8		12.0
		(42.2-49.5)		(57.6-64.0)		(50.1-57.9)		(5.7-9.9)		(9.5-14.5)
Work status										
Not working (reference)		47.8		65.5		55.4		7.0		12.4
		(45.7-49.9)		(63.4-67.6)		(53.3-57.4)		(6.0-8.1)		(11.0-13.8)
Working		49.8		65.2		54.7		8.3		14.2*
		(48.3-51.4)		(63.7-66.7)		(53.3-56.2)		(7.4-9.2)		(13.1-15.3)
Age										
15-24 (reference)		49.4		66.2		47.3		5.8		13.2
		(44.9-53.8)		(61.8-70.6)		(43.1-51.5)		(6.8-8.5)		(10.3-16.0)
25-34		50.1		65.5		54.8**		7.2		14.2
		(47.7-52.4)		(63.3-67.7)		(52.5-57.2)		(6.1-8.3)		(12.6-15.7)
35-44		47.1		64.5		57.4**		8.6		13.0
		(44.4-49.8)		(62.0-67.1)		(54.7-60.1)		(7.1-10.1)		(11.1-14.9)
45-49		51.5		65.4		59.8**		11.3*		13.9
		(46.8-56.2)		(61.2-69.7)		(55.1-64.4)		(8.2-14.5)		(10.4-17.3)
Marital status										
Married (reference)		49.6		65.3		54.6		7.7		13.3
		(48.0-51.3)		(63.5-67.1)		(43.0-56.2)		(6.8-8.5)		(12.2-14.5)
Cohabiting		48.4		65.3		54.7		8.1		13.9
		(46.3-50.4)		(63.1-67.5)		(54.5-57.5)		(6.9-9.4)		(12.4-15.4)
Partner’s age										
14-24 (reference)		50.5		67.2		54.8		9.7		18.8
		(44.9-56.2)		(62.0-72.4)		(49.6-60.0)		(6.2-13.3)		(13.8-23.9)
25-34		49.5		65.3		53.0		8.2		13.7*
		(46.8-52.2)		(62.9-67.8)		(50.2-55.8)		(6.5-9.9)		(11.8-15.6)
35-44		47.4		64.2		55.5		8.0		12.5*
		(47.1-52.0)		(62.0-66.4)		(53.3-57.7)		(6.9-9.1)		(10.9-14.0)
45+		47.4		66.1		57.3		6.8		12.7
		(43.4-51.5)		(62.2-69.9)		(53.4-61.2)		(5.1-8.5)		(10.1-15.3)
Partner’s age, relative to respondent										
Same age or younger (ref.)		50.3		65.9		57.2		6.8		14.3
		(47.7-52.9)		(63.4-68.4)		(54.8-59.6)		(5.6-8.0)		(12.6-16.0)
1-5 years older		49.5		65.9		53.7*		7.7		12.8
		(47.6-51.4)		(64.0-67.7)		(51.9-55.4)		(6.7-8.7)		(11.6-14.0)
6+ years older		47.4		63.7		54.6		9.4*		14.0
		(44.7-50.1)		(61.2-66.3)		(51.7-57.5)		(7.7-11.0)		(12.1-16.0)
Partner’s education										
None (reference)		47.9		63.7		53.2		7.0		16.9
		(38.8-57.0)		(54.7-72.7)		(43.9-62.4)		(1.8-12.2)		(8.3-25.6)
Primary		50.1		65.7		55.4		8.7		14.7
		(47.6-52.7)		(63.5-67.8)		(53.0-57.8)		(7.4-10.0)		(13.0-16.4)
Secondary		50.7		66.5		55.1		7.0		12.7
		(48.5-52.8)		(64.3-68.8)		(53.0-57.1)		(6.0-8.1)		(11.1-14.3)
Higher		43.8		62.3		53.8		7.0		12.3
		(39.5-48.1)		(58.6-66.1)		(49.7-58.0)		(5.0-9.1)		(9.4-15.1)
Partner’s education compared to respondent										
Wife is more educated (reference)		47.9		63.6		55.2		7.5		12.2
		(44.9-50.9)		(60.9-66.4)		(52.4-58.0)		(5.9-9.0)		(10.3-14.1)
Same level of education		46.9		63.5		54.6		6.7		13.8
		(44.4-49.4)		(61.0-65.9)		(52.1-57.0)		(5.4-8.0)		(12.0-15.5)
Husband is more educated		50.6		66.8		55.0		8.4		14.1
		(48.7-52.6)		(65.0-68.6)		(53.1-56.9)		(7.3-9.5)		(12.7-15.4)

*p < 0.05 **p < 0.01 ***p < 0.001.

Ψ Respondents who experienced sexual abuse may also have experienced psychological and/or physical abuse.

### Depression

The unadjusted results shown in Table 2 indicate that the percentage of women who report having symptoms of depression varies according to their exposure to intimate partner violence. The adjusted percentages show that these differences persist after controlling for other factors. After controls, the percentage of women who report feeling tired all the time remains significantly higher among women experienced psychological abuse only (57.5%; p < .001), who experienced physical abuse (55.5%; p < .01), and those who experienced sexual abuse (66.4%; p < .001).

The results for ‘difficulty doing daily activities’ and ‘difficulty making decisions’ show nearly identical patterns. That is, psychological abuse and physical abuse have nearly equal effects, while sexual abuse has the strongest effect. The results further suggest that women who were physically or sexually abused by their partner are much more likely than unabused women to report that they cry easily (78.6% and 85.9%, respectively, vs. 72.7%; p < .001). The adjusted percentages show that these effects cannot be explained by the respondent’s socioeconomic status, her relative age and education compared to her partner, or by experiences with abuse in her parental household.

Childhood experience with abuse in the parental household is also associated with the risk the women’s report experiencing symptoms of depression, but the effects are not consistent. Women who report experiencing physical abuse in childhood are more likely than other women to report crying easily (76.2% vs. 72.1%; p < .001). However, experiencing physical abuse in childhood is not associated with other symptoms of depression. In addition, women who report that their father beat their mother, or who report they were unsure about it, are more likely than other women to cry easily (76.7% vs. 73.2%; p < .01) and more likely to have difficulty making decisions (50.1% vs. 44.1%; p < .001).

### Anxiety

The prevalence of symptoms of anxiety varies depending on the respondent’s exposure to domestic abuse in the past year. For example, after controls, 46.0% of women who report not being abused by their partner report having feelings of fear without apparent reason. This percentage increased to 49.7% (p < .05) for those women who experienced psychological abuse and to 53.5% (p < .001) for those who experienced physical abuse. Among women who were sexually abused, 59.3% express having feelings of fear (p < .001). It is noteworthy that psychological abuse and physical abuse by a partner are both associated with significantly higher scores on all three anxiety indicators. However, psychological and physical abuse have nearly identical effects on ‘scaring easily’ and ‘having headaches of great intensity.’ By contrast, physical abuse has a stronger effect than psychological abuse on the likelihood that women have feelings of fear for no apparent reason. Women who were sexually abused by their partner have the highest scores on all three indicators of anxiety.

Childhood exposure to domestic violence is not associated with having symptoms of anxiety. Although women who were physically abused in childhood are slightly more likely than other women to report having headaches of great intensity (56.1% vs. 52.1%; p < .001), there is no effect on the other two indicators of anxiety. Psychological abuse in childhood, or having witnessed spousal abuse in the parental household are not associated with any of the three symptoms of anxiety.

### Psychogenic non-epileptic seizures

The percentage of women who reported having convulsions or attacks with tongue biting is associated with intimate partner violence. The adjusted percentage of women who report having convulsions or attacks with tongue biting is highest among women who experienced sexual abuse (12.4%; p < .001), followed by physical abuse (9.4%; p < .001) and psychological abuse (8.6%; p < .01). Having experienced physical or psychological abuse in childhood is not associated with the likelihood that women report having had convulsions. Three regions stand out because of their high prevalence of convulsions: Cochabamba (11.3%), Beni (11.6%), and Pando (12.9%).

### Psychotic disorders

Intimate partner violence is associated with hearing voices that others do not hear. The percentage of women who reported hearing voices is significantly higher among women who experienced sexual abuse (24.0%; p < .001) or physical abuse (15.3%; p < .01) but not among women who experienced only psychological abuse. Women from Tarija and Pando are more likely to report such symptoms.

## Discussion

The results of this study confirm that intimate partner violence is common in Bolivia. These findings are consistent with a large body of literature that shows that intimate partner violence is common in Latin America [1-5]. Our data show that overall, nearly one in two women in Bolivia (47%) experienced some type of intimate partner violence in the past year. Nineteen percent of women in union report being physically abused by their partner and seven percent were sexually abused by him. Most victims of sexual abuse were also physically and/or psychologically abused. Moreover, one in five Bolivian women (21%) reported that they were psychologically abused by their partner, although they were not physically or sexually abused. A recent review article on the health implications of IPV noted that the majority of studies also found that women often experience more than one type of IPV [55].

As noted elsewhere, there is a need for further research on the implications of different types of IPV and of experiencing multiple types of IPV [55]. Existing studies on the association between IPV and health outcomes often examine IPV as a single construct, without distinguishing between different types of IPV. The high prevalence of psychological abuse by intimate partners (in absence of either physical or sexual abuse) that we have observed in the Bolivia data highlights the need to study not only the potential adverse effects of different types of IPV on mental health, but to also examine whether experienced *only* psychological abuse affects mental health. Although numerous studies have examined the relationship between IPV and mental health in the developed world, our study is one of the few population-based studies that examines this association in a developing country (for other examples, see [16,17]).

Our findings support findings from several other studies – mostly from the U.S. – that show that intimate partner violence is associated not only with physical health, but also with mental health [56-61]. Studies on the association between intimate partner violence and mental health among Latina women in the U.S. indicate that experience with intimate partner violence is associated with adverse mental health effects [62-64]. Our data from Bolivia show that women who experienced intimate partner violence are more likely than other women to experience symptoms of mental health disorders.

While nearly all existing studies examine the combined effects of physical, psychological, and sexual violence on health outcomes, our study distinguishes between these three types of partner abuse. Moreover, we also specifically examined the effect of having experienced *only* psychological IPV. Because there are indications that the total IPV disease burden is caused mostly by depression and to a lesser extent by anxiety [50,55], our study separately examined the association between different types of IPV and symptoms of depression, anxiety, psychogenic seizures, and psychotic disorders, rather than using a single mental health construct as has been done in some other studies [16,17].

As anticipated, our analyses show that Bolivian women who experienced physical abuse by their intimate partner in the last year are much more likely to experience symptoms of depression, anxiety, psychogenic seizures, and psychotic disorders, irrespective of their childhood experiences with domestic abuse, their social and economic status, or partner and couple characteristics. Women who were sexually abused by an intimate partner are even more likely to experience undesirable mental health outcomes. It is noteworthy that most victims of sexual abuse by their partner were also physically or psychologically abused. This combination of different types of abuse is likely to have exacerbated these negative mental health outcomes. These findings are consistent with other studies of the effect of different types of IPV on symptoms of mental health problems. It also reinforces earlier findings that experiencing multiple types of IPV increases the likelihood of probability of experiencing symptoms of depression and post-traumatic stress disorder [16,55].

Another important finding of our study is that for Bolivian women, experiencing psychological abuse is also associated with an increased risk of experiencing symptoms of depression, anxiety, or psychogenic seizures. In fact, women who *only* experienced psychological abuse by their intimate partner are nearly as likely as women who were physically abused to report having symptoms of depression, anxiety, and psychogenic seizures. A study of Spanish women also found that psychological IPV had a similar effect on symptoms of depression as physical IPV [65]. Our study did not find any evidence that psychological abuse alone is associated with an increased prevalence of symptoms of psychotic disorders. These findings are consistent with U.S. studies that found that physical, emotional, and sexual intimate partner violence were each associated with mental health problems, including depression [59,60,66,67].

## Conclusions

Our findings show that there is an urgent need for studies on the prevalence and health consequences of psychological intimate partner violence in other developing countries. Because evidence is building that IPV-related mental health problems are particularly acute among Latina women [62-64], more research on Latin American countries is particularly warranted. The finding that women who *only* experienced psychological abuse by an intimate partner report have nearly identical symptoms of mental health problems as women who were physically abused has important programmatic implications. Specifically, it highlights the importance of ensuring that victims of intimate partner violence have adequate access to mental health services. Research on Hispanic women in the U.S. has also emphasized the need for culturally appropriate outreach programs to increase awareness of the negative effects of intimate partner violence on women’s mental health and to increase women’s awareness about how to access mental health services [63]. The finding that physical abuse and psychological abuse are often experienced concurrently further suggests that health providers who treat victims of physical intimate partner violence should also screen these patients for symptoms of potential mental health problems and – if needed – refer them to appropriate mental health services.

## Competing interests

The authors declare that they have no competing interests.

## Authors’ contributions

DM and SP conducted the data analysis and prepared the first draft of the manuscript. PH contributed to the analysis plan and made critical revisions to the methodology. All authors read and approved the final manuscript.

## Pre-publication history

The pre-publication history for this paper can be accessed here:

http://www.biomedcentral.com/1472-6874/13/28/prepub
